# Supplementary material for: MetaQTL: a package of new computational methods for the meta-analysis of QTL mapping experiments
Source: BMC Bioinformatics. 2007 Feb 8;8:49. doi: 10.1186/1471-2105-8-49 (PMC1808479; doi:10.1186/1471-2105-8-49)
Supplement: Additional File 7 — MetaQTL Package : jar file and tutorial. This Zip archive contains both the MetaQTL JAR file and the files of the tutorial. [file 1471-2105-8-49-S7.zip › org.inra.metaqtl/doc/org/metaqtl/Qtl.QTLSupport.html]

Qtl.QTLSupport


|  |  |  |
| --- | --- | --- |
| **Overview** \| **Package** \| **Class** \| **Use** \| **Tree** \| **Deprecated** \| **Index** \| **Help** | |  |
| **PREV CLASS**   **NEXT CLASS** | **FRAMES**    **NO FRAMES**     **All Classes** |  |
| SUMMARY: NESTED \| FIELD \| CONSTR \| METHOD | DETAIL: FIELD \| CONSTR \| METHOD |  |


---


## org.metaqtl Class Qtl.QTLSupport

```
java.lang.Object
  org.metaqtl.Qtl.QTLSupport
```

**All Implemented Interfaces:**: java.io.Serializable

**Enclosing class:**: Qtl

---

``` public class Qtl.QTLSupport extends java.lang.Object implements java.io.Serializable ```

This class stores information
on the QTL Confidence Interval

**See Also:**: Serialized Form

---

| **Field Summary** | |
| --- | --- |
| `double` | `from`             The position from the first marker to the left border of the IC. |
| `double` | `ic1`             The recombination rate between the QTL position and the left border of the IC. |
| `double` | `ic2`             The recombination rate between the QTL position and the right border of the IC. |
| `double` | `lodDecrease`             The value of the LOD decrease used to obtain the CI. |
| `double` | `to`             The position from the first marker to the right border of the IC. |


**Constructor Summary**

`Qtl.QTLSupport()`


| **Method Summary** | |
| --- | --- |
| `Qtl.QTLSupport` | `getCopy()` |

**Methods inherited from class java.lang.Object**

`clone, equals, finalize, getClass, hashCode, notify, notifyAll, toString, wait, wait, wait`

**Field Detail**

### ic1

```
public double ic1
```

:   The recombination rate between the QTL position
    and the left border of the IC.

---


### ic2

```
public double ic2
```

:   The recombination rate between the QTL position
    and the right border of the IC.

---


### from

```
public double from
```

:   The position from the first marker
    to the left border of the IC.

---


### to

```
public double to
```

:   The position from the first marker
    to the right border of the IC.

---


### lodDecrease

```
public double lodDecrease
```

:   The value of the LOD decrease used to obtain the CI.


**Constructor Detail**

### Qtl.QTLSupport

```
public Qtl.QTLSupport()
```


**Method Detail**

### getCopy

```
public Qtl.QTLSupport getCopy()
```

:   **Returns:**


---


|  |  |  |
| --- | --- | --- |
| **Overview** \| **Package** \| **Class** \| **Use** \| **Tree** \| **Deprecated** \| **Index** \| **Help** | |  |
| **PREV CLASS**   **NEXT CLASS** | **FRAMES**    **NO FRAMES**     **All Classes** |  |
| SUMMARY: NESTED \| FIELD \| CONSTR \| METHOD | DETAIL: FIELD \| CONSTR \| METHOD |  |


---
